# On the systematic position of *Baltimartyria* Skalski, 1995 and description of a new species from Baltic amber (Lepidoptera, Micropterigidae)

**DOI:** 10.3897/zookeys.130.1480

**Published:** 2011-09-24

**Authors:** Wolfram Mey

**Affiliations:** 1 Museum für Naturkunde, Leibniz Institute at the Humboldt Universität, Invalidenstr. 43, D – 10115 Berlin

**Keywords:** Insecta, Lepidoptera, Micropterigidae, fossils, Baltic Amber

## Abstract

This paper describes a rare case of a male moth in Baltic amber in an excellent position for establishing a species. The moth represents the second species of the genus *Baltimartyria* Skalski, 1995, described herein as *Baltimartyria rasnitsyni* **sp. n.** The detection of this new species prompts research on the systematic position of the genus within the family Micropterigidae. The genus was found to provide none of the apomorphic characters that would allow placement in one of the monophyletic lineages within the family. The genus is provisionally assigned to the “southern sabatincoid group”, a weakly supported assemblage of Southern Hemisphere genera. The sister genus has still to be determined. *Baltimartyria* is the first North Hemisphere representative in this group. Some general aspects of historical biogeography relevant for the group are briefly discussed.

## Introduction

In comparison with other mega-diverse insect orders preserved in Baltic amber, the Lepidoptera are poorly known (Kristensen and Skalski 1998, Rasnitsyn and Quicke 2002). The reason for this inadequate knowledge is not the rarity of inclusions, but rather the main morphological feature of lepidopterans – the scales. They cover the whole body and its appendages and hide important diagnostic characters, e. g. wing venation. The scaling provides, however, traits like colour patterns, but these are barely preserved and discernable in amber specimens. The wings are often folded and may frequently obscure features such as the abdomen and its copulatory structures. In many groups the genitalia are also retracted within the distal abdominal segments. Therefore, lepidopterans can rarely be identified at the species level and even assignment to a discrete family may often not be feasible. This applies to the majority of amber Lepidoptera which are usually dominated by micro moths. Only in cases where wings are outspread and genitalia visible can specimens potentially be determined to species.

For many years I had a Baltic amber piece on my desk containing a male moth in a splendid position. The up-held and slightly rubbed wings reveal venational and partial genitalic characters well. The species clearly belongs to Micropterigidae, the most ancestral family of extant Lepidoptera. A number of fossil genera and species were described and assigned to this family (Koslov 1988, Koslov et al. 2002) but their placement in Micropterigidae was rejected by Kristensen and Skalski (1998) because phylogenetically important details were not preserved. Some micropterigid moths described from amber are true and undisputed representatives of the family. The few, hitherto described and named taxa are listed in Table 1 (see Nieukerken et al. in press for the most up-to-date overview of micropterigid fossils and their taxonomic placement). Some further specimens are known, but remained undescribed and unnamed (e.g. Grimaldi and Engel 2005: 562). Considering only described species, the Baltic amber fauna is apparently very poor in individuals of this family. To date, only three inclusions have been reported belonging to three species: *Micropterix proavitella* Rebel, 1936, *Electrocrania immensipalpa* Kuznetzov, 1941 and *Micropterix gertraudae* Kurz & Kurz, 2010. From amber collected in western France only some scales of a presumed but unnamed micropterigid species were discovered and described by Kühne et al. (1973).

**Table 1.** Records of Micropterigidae described from amber.

**species**	**origin**	**first revision**	**current combination**
*Micropteryx pervetus* Cockerell, 1919	Burmese amber	Whalley (1978): *Sabatinca*	*Sabatinca* s.l.
*Micropteryx proavitella*Rebel, 1936	Baltic amber	Skalski (1995):*Baltimartyria*	*Baltimartyria*
*Parasabatinca aftimacra*Whalley, 1978	Lebanese amber		*Parasabatinca*
*Micropterix gertraudae*Kurz & Kurz, 2010	Baltic amber		*Micropterix*

Kozlov (1988: 26) synonymised *Electrocrania* Kuznetzov, 1941 with *Micropterix* Hübner, 1815, but this was rejected by Kristensen and Skalski (1998) because of the presence of a mesotibial spur. The holotype of *Micropterix proavitella* was examined by Skalski (1995) who confirmed the placement of the species in Micropterigidae on the basis of wing venation, head morphology, greatly shortened labial palps, the absence of mesotibial spurs and the desclerotised sternum of abdominal segment VIII. However, he realised that the species is not a member of *Micropterix* but represents a separate genus for which he introduced the new name *Baltimartyria* Skalski, 1995. Skalski`s (1995) detailed re-description and illustration of the holotype facilitated the identification of the individual in my possession. At a first glance, it was thought to represent a second specimen of *Baltimartyria proavitella*, becauseof its similar wing pattern, venation and valvae. A closer inspection, however, revealed some clear differences important enough to regard the specimen as a distinct species, which is described below.

## Taxonomy

### Family Micropterigidae. Baltimartyria Skalski, 1995

#### Baltimartyria rasnitsyni sp. n.

urn:lsid:zoobank.org:act:1D69948C-B1FF-44BC-B313-2BAA38FA82DF

http://species-id.net/wiki/Baltimartyria_rasnitsyni

**Figures 1–2.**
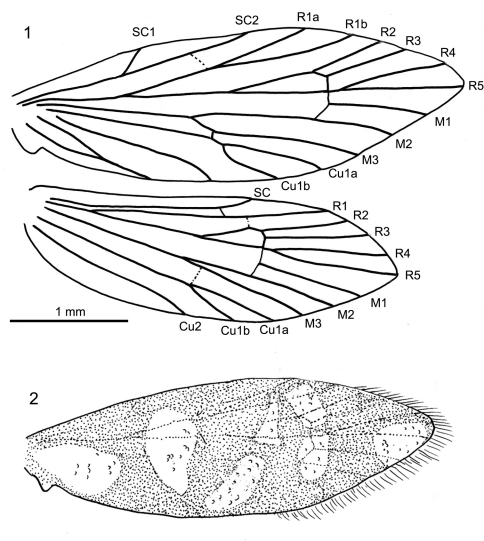
*Baltimartyria rasnitsyni* sp. n. **1** wing venation **2** reconstructed wing pattern.

Figs 1 [Fig F2] [Fig F3] [Fig F4] 11

##### Material. 

Holotype male, Baltic Amber, MB.I 5950, deposited in Museum für Naturkunde, Berlin.

##### Preservation.

The adult moth is completely preserved and clearly visible from a ventro-lateral view. Right maxillary palps and antenna covered by body, dorsal and inner side of genitalia filled by white emulsion.

##### Etymology.

Named in honour of Alexandr P. Rasnitsyn, the eminent Russian paleoentomologist.

##### Diagnosis.

*Baltimartyria rasnitsyni* sp. n. can be separated from *Baltimartyria proavitella* by segment four of maxillary palps being as long as third and second segment together, and by shortly stalked R4 and R5 in both fore- and hindwings. In *Baltimartyria proavitella*, the two terminal segments of the maxillary palps are as long as the third segment, and in the fore- and hindwings all terminal R branches originate separately from the cell.

##### Description.

Length of forewing 4 mm, length of body 3 mm; head with erect, piliform scales on vertex, ocelli pale white, scape and pedicel together as long as eye diameter, scaled, 35 barrel-shaped flagellomeres present, basal segments (1–3) with scales, subsequent segments unscaled, each flagellomere with two whorls of long sensilla trichodea, one basal and one at mid-length, ascoids not clearly visible; maxillary palps five segmented, basal segments of equal length, fourth segment longest, terminal segment short, acute and with short bristles; labial palps two-segmented, terminal segment small, rounded; mandibles present (only base visible); fore-tibia with blade-like epiphysis exhibiting an acute tip, spurs 0.0.4, basitarsus of all legs covered with short, acute and semi-erect scales, tarsal segments with terminal, pair of ventral bristles; reconstructed wing pattern in Fig. 2, venation in Fig. 1, R1 with two branches in forewing, R4 and R5 on a short common stalk from cell in both wings, (anal field of hindwing only partially visible); ventral side of abdominal segment VIII membranous (= pale brown), lateral sides sclerotised (= dark brown), vinculum deeply retracted into segment VIII (segmental limits obsolete by milky nebulae); valvae simple, elliptical and spoon-like, outer surface covered by hairs.

**Figures 3–8. F2:**
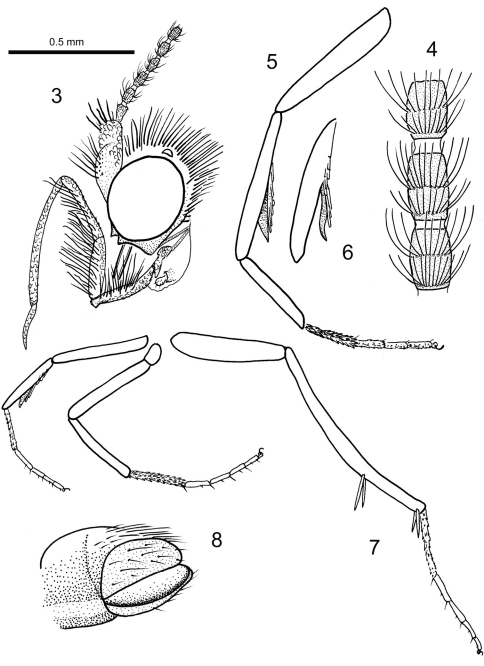
*Baltimartyria rasnitsyni* sp. n. **3** head, lateral view **4** flagellomeres from mid-antenna, enlarged **5** foreleg, enlarged **6** tibia of foreleg with epiphysis from different view **7** legs **8** tip of abdomen and genitalia, ventrolateral view.

**Figure 9. F3:**
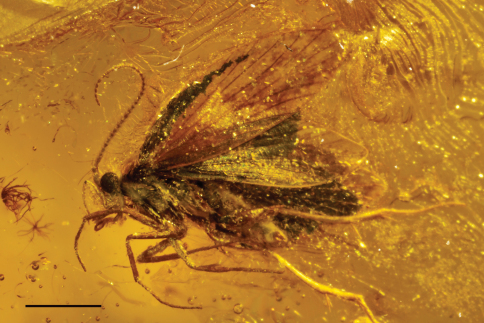
Male holotype of *Baltimartyria rasnitsyni* sp. n. Right hand side position, scale bar 1 mm.

**Figure 10. F4:**
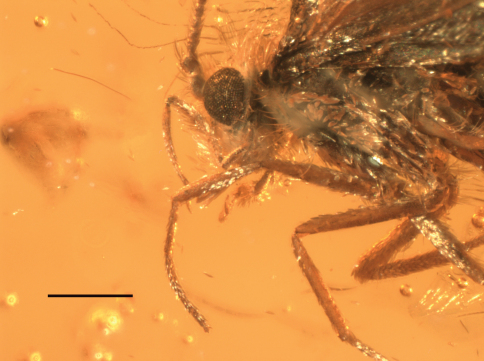
Male holotype of *Baltimartyria rasnitsyni* sp. n. Details of head, scale bar 1 mm.

**Figure 11.**
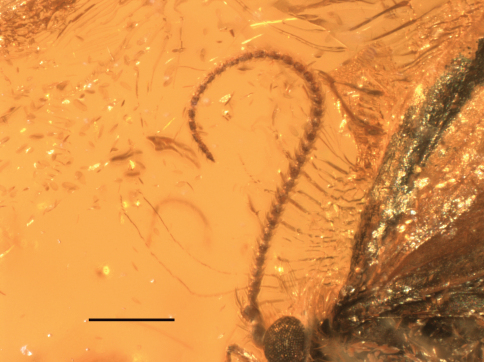
Male holotype of *Baltimartyria rasnitsyni* sp. n. Antenna, scale bar 1 mm.

## Systematic position

**Figure 12.**
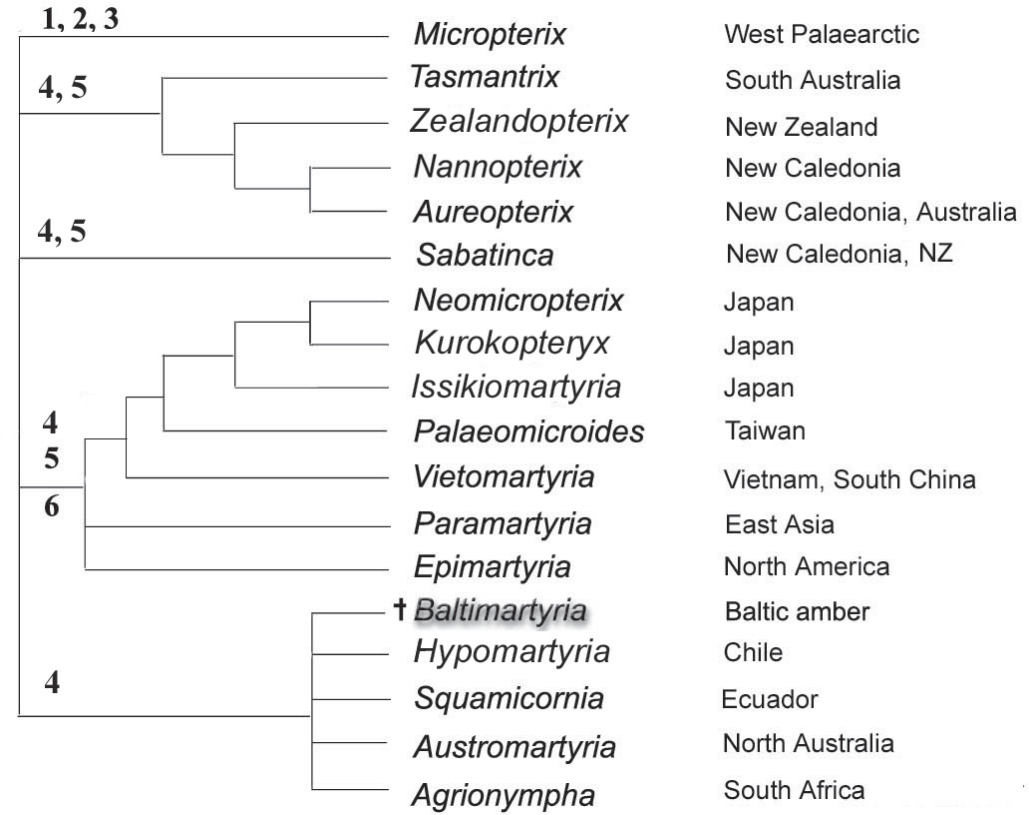
Presumed phylogenetic relationships within Micropterigidae based on Gibbs (2010).

Skalski (1995) assigned *Baltimartyria* to the so-called *Sabatinca* group of genera on the basis of genital structures and wing venation. The concept of the *Sabatinca* group by 1995 is now obsolete, and the *Sabatinca* group of Gibbs (2010) is more restricted. He noted a resemblance with the Chilean genus *Hypomartyria* (Kristensen and Nielsen 1982) but did not suggest any synapomorphies which could be regarded as evidence of a close relationship. Recently, most genera of the former *Sabatinca* group were revised (Gibbs 2010, Gibbs and Kristensen 2011, Hashimoto 2006, Imada et al. 2011). The phylogenetic significance of a number of morphological characters was re-evaluated and new characters were found to be useful for reconstructing phylogenetic relationships. A molecular analysis of the 16S rRNA gene provided evidence for the separation of Micropterigidae into five monophyletic lineages (Gibbs et al. 2004). Based on the description of this phylogeny (Gibbs 2010: 3) which includes a hypothesized ‘Australian group’ comprising four genera (Gibbs 2010: 43, fig. 16) a cladogram was constructed (Fig. 12) which, the modest resolution notwithstanding, suffices for the present discussion. Since *Baltimartyria* can be excluded from the *Micropterix* branch, the question arises, where does the genus fit in this topology? Fossil taxa usually have a restricted set of characters which can be used for placing them together with extant species in a phylogenetic tree. Six informative characters, which can be observed in *Baltimartyria*, were identified. They are described in Table 2, and the distribution of their apomorphic states is indicated by the corresponding numbers in the cladogram of Fig. 12. Hindwing venation (5) and modified antennae (6) exclude the genus from three lineages. The remaining lineage, the “southern sabatincoid group” (comprising *Hypomartyria*, *Austromartyria* and *Agrionympha*; the assignment of *Squamicornia* to this assemblage is conjectural) is based on resemblances in the structure of sternum V gland protuberances, presence of both dorsal and ventral valve muscles from segment IX and morphology of early instar larvae. These characters may be plesiomorphic within the family (Gibbs and Kristensen 2011). Unfortunately, the abdomen of the holotype of *Baltimartyria rasnitsyni* sp. n. is covered by numerous hairs that do not allow discerning structures on segment V. This was the only character which could be observed on fossil specimens. Thus, no apomorphies have been identified which would support the monophyly of this assemblage. But only this weakly supported group yields no characters conflicting with placement of *Baltimartyria* within. Therefore, the genus is here provisionally assigned to the “southern sabatincoid group”. The sister genus of *Baltimartyria* thus remains to be identified. According to wing venation, the “southern sabatincoid group” members have largely retained the plesiomorphic states ascribed to the Lepidoptera ground plan (Kristensen 1984). All other micropterigid lineages have developed a few apomorphic characters in the wings. It seems quite likely therefore that this “southern sabatincoid group” contains the extant genus which is most overall similar to the last ancestor of the family.

**Table 2.** Characters of Micropterigidae observable in *Baltimartyria* and relevant for its placement.

**number**	**character**	**plesiomorphic state**	**apomorphic state**
1	R in forewing	all branches to costa	R5 to wing apex
2	R1 in forewing	forked	simple, unforked
3	male segment IX	caudal margin simple and straight	caudal margin with processes
4	male venter VIII	sclerotised	more or less membranous
5	R in hindwing	complete vein to costal margin	more or less coalescent with Sc
6	antenna	filiform, flagellomeres barrel-shaped	flagellomeres modified

## Historical biogeography

Given the uncertainty about the placement of *Baltimartyria* any discussion on aspects of its historical biogeography is premature. However, the following, general remarks might be useful for considering in future discussions.

According to the geographic distribution of extant Micropterigidae (Gibbs 1983), *Baltimartyria* was initially expected to belong to the Northern Hemisphere genera of the *Sabatinca* group. Morphological characters, however, suggest a placement in an assemblage comprising Southern Hemisphere genera. The distribution pattern of these Southern genera points to a Gondwanan origin of the ancestor of the group and its subsequent splitting into several evolutionary lines, following the disintegration of the Gondwana palaeocontinent. However, if the “southern sabatincoid group” including *Baltimartyria* is indeed a monophylum, this hypothesis would be challenged, and the notion of a world-wide distribution prior to the splitting of Gondwana favoured. Eskov (2002) has compiled and discussed similar examples of alleged South Hemisphere species (in Plecoptera, Mecoptera, Diptera, etc.) found as fossils in Eurasia and concluded, that many of these presumed Gondwana elements had a much wider distribution in earth history but eventually survived in refuge areas in Southern Hemisphere continents only. Thus, their Gondwanan origin is questionable.

However, in Eskov`s discussion on Gondwanan vs. non-Gondwanan origin of taxa the role of drifting terranes is not considered. In South East Asia a series of terranes were identified, which were attached to the Asian continent during the Paleozoic and Mesozoic. They all arrived from the south and had their origin on the northern margin of Gondwana (Metcalfe 1998, 2001, Hall 2001). A permanent, sub-aerial drift of these terranes, inhabited by plant and animal species, allowed their survival during the passage and is considered to have imported them finally into the Asian biota. This passive transport is one of the mechanisms which has contributed to a wider distribution of taxa in the Mesozoic and Cenozoic. During the passage from southern to northern latitudes the biota on drifting terranes had to cope with a change in climatic conditions. Crossing the equator and the Inter-Tropical Convergence Zone in the Mesozoic was probably not as dramatic as it was today because of a generally warmer climate and less pronounced temperature gradient along latitudes. Nonetheless, species had to adapt to changing climatic and ecological conditions. The presence of mountain ranges on terranes could have facilitated survival by vertical shifts of distribution ranges. The Mesozoic equatorial crossing of Gondwanan terranes certainly affected the biota in several ways. Its significance can be investigated today by comparing the distribution of endemic taxa in Madagascar and India. India collided with Asia in the early Tertiary (Hall 2001). In the Mesozoic the Indian plate was united with Madagascar. The recent discovery of Micropterigidae in Madagascar allows the inference, that the family was present on the Indian plate as well. If species survived the northward drift they evolved in isolation and should be today the closest relatives of the Madagascan taxa. Up to now species of the *Sabatinca* group were not found to occur on the Indian subcontinent. Either they became extinct, or they escaped discovery until now. A distinctive clade of *Micropterix* has been described for India (Lees et al. 2010) but is attributed to the Palearctic radiation on the basis of morphological and molecular evidence. Extinction of the *Sabatinca* group might point to the significance of changing conditions during drift. Discovery in India is plausible since the species on Madagascar were detected only recently (Lees et al. 2010), despite intensive research on Lepidoptera (including microlepidopterans) in the decades before.

There are undoubtedly more undetected micropterigid species, fossil and extant, on the globe. Each discovery provides new information and throws new light on current phylogenetic and biogeographic reconstructions.

## Supplementary Material

XML Treatment for Baltimartyria rasnitsyni
